# Changing climate change: The carbon budget and the modifying-work of the IPCC

**DOI:** 10.1177/0306312720941933

**Published:** 2020-07-16

**Authors:** Bård Lahn

**Affiliations:** 1 TIK Centre for Technology, Innovation and Culture, CICERO Centre for International Climate Research, University of Oslo, Norway

**Keywords:** carbon budgets, IPCC, issue formation, modifying work, politics of climate change, problematization

## Abstract

Over the last 10 years, the concept of a global ‘carbon budget’ of allowable CO_2_ emissions has become ubiquitous in climate science and policy. Since it was brought to prominence by the Fifth Assessment Report of the IPCC, the carbon budget has changed how climate change is enacted as an issue of public concern, from determining the optimal rate of future emissions to establishing a fixed limit for how much emissions should be allowed before they must be stopped altogether. Exploring the emergence of the carbon budget concept, this article shows how the assessment process of the IPCC has offered scientific experts the means to modify how the climate issue is problematized, and discusses the implications of this ‘modifying-work’ for the politics of climate change. It finds that the ‘modified climate issue’ must be seen as an outcome of the ordinary work within established scientific and political institutions, and the agency these institutions afford scientists to enact the issue differently. On this basis, it argues that the case of the carbon budget holds important insights not only for the relationship between climate science and policy, but also for the pragmatist literature on ‘issue formation’ in STS.

## Introduction

What kind of issue is ‘the climate issue’? The question is brought to the fore by the growing interest in the ‘issue-centric’ approach to studying publics and politics in Science and Technology Studies (STS). In recent years, the role of issues in mediating public participation around objects or material entanglements has been highlighted as deserving particular attention in STS analysis (Callon, 2009; Latour, 2007; Marres, 2005, 2007). While this literature frequently discusses climate change as an example of such an issue (e.g. Asdal, 2014; Blok, 2014; Marres, 2012), it has also highlighted how the climate issue is enacted in multiple and shifting ways, raising problems ranging from the geopolitical to the personal, and from urgent questions of North/South justice to the long-term challenge of understanding the role of humans in geological history. Thus, the ‘highly uneven and shape-shifting’ ways in which climate change is enacted (Blok, 2014: 47) makes it exceedingly difficult to answer the seemingly simple question of what kind of issue we are, in fact, faced with.

What may be STS’s most important contribution to answering the above question has been to point out how climate change is defined in ‘intensely scientific’ terms (Wynne, 2010: 291). A large literature has shown how climate change has been co-produced as an issue of political and scientific concern (Demeritt, 2001; Mahony and Hulme, 2018; Miller, 2004). This includes how climate modelling and physical climate science has been instrumental in constituting ‘global climate’ and ‘future climate’ as knowable and governable entities (Edwards, 2010; Lahsen, 2007; Shackley, 2001). It also includes, albeit to a lesser extent, the role of economics in articulating the central scientific and political problem of achieving ‘climate stabilization’ at optimal levels – that is, determining the exact concentration of greenhouse gases that ensures an economically optimal balance between the changes in the climate system following from greenhouse gas emissions, and the costly changes in social systems required to limit those emissions (Boykoff et al., 2009; Lohmann, 2009; Randalls, 2011).

The view of the climate issue as a problem of stabilizing the global climate at some future ‘optimal’ level was institutionalized in the 1980s and ’90s, through the establishment of the Intergovernmental Panel on Climate Change (IPCC) and the UN Framework Convention on Climate Change (UNFCCC). As the globally authoritative scientific and political bodies to which the issue is delegated, the IPCC and the UNFCCC can be seen as the institutional expression of the historical process of co-production described by STS scholars (e.g. Miller, 2004; Yearley, 2009): The objective of the UNFCCC is to achieve a ‘stabilization of greenhouse gas concentrations in the atmosphere at a level that would prevent dangerous anthropogenic interference with the climate system’ (Boykoff et al., 2009; Oppenheimer and Petsonk, 2005), while the voluminous assessment reports of the IPCC have provided scientific knowledge about the levels at which concentrations should be stabilized (Hulme and Mahony, 2010; van der Sluijs et al., 1998). The concept of ‘climate stabilization’, in other words, has itself been implicated in efforts to ‘stabilize’ a specific enactment of the climate issue.

But while the institutional set-up of the UNFCCC and the IPCC has proven durable over several decades, their historical enactment of climate change in terms of ‘climate stabilization’ appears to be less so. Over the past few years, for example, a wave of school strikes and high-profile protests around the world have sketched the contours of a climate issue that seems far removed from the one enacted by the international climate governance institutions of the 1990s: Activist demands to declare a ’climate emergency’ and to leave fossil fuels in the ground differ starkly from previously dominant ideas of gradual and optimal management of atmospheric greenhouse gas concentrations based on cost-benefit analysis and multilateral diplomacy. In this way, the school strikes and similar calls for emergency climate action seem to pose a radical challenge to the traditional ‘climate stabilization’ approach of the UNFCCC and the IPCC. They thereby also illustrate how pinning down the precise boundaries of a stabilized ‘climate issue’ remains inherently difficult.

This article contributes to understanding how climate change changes. More specifically, I analyse ongoing modifications in how climate change is enacted as a matter of public concern. My entry point is an in-depth study of an important modification of the climate issue that has taken place over the last decade, in which the previously dominant concept of ‘climate stabilization’ has been challenged and partly replaced by the notion of a global ‘carbon budget’.

The carbon budget first appeared as a central concept in the IPCC’s Fifth Assessment Report, published in 2013–2014. This report no longer enacted the climate issue primarily in terms of the atmospheric concentrations and long-term stabilization levels associated with the ‘climate stabilization’ concept. Rather, it highlighted *the cumulative amount of carbon emitted from human activities* as the relevant metric for climate policymaking. The report thus changed the core scientific and political problem of climate change from determining the optimal rate of future emissions to establishing a fixed limit – a budget – for how much emissions should be allowed before they must be stopped altogether. This move had important implications for climate politics, for example, by underwriting calls to phase out or reach ‘net zero’ carbon emissions, by sharpening disagreement over distributive justice in international climate diplomacy, and by underpinning a surge of activism to keep fossil fuels in the ground.

The emergence of the carbon budget fuelled new controversies in climate politics and strengthened the radical demands put forward in recent school strikes and protests against fossil fuel projects. However, while it may be tempting to read this as a story of external activists challenging how existing institutions seek to know and govern the global climate, the core claim of this article is the opposite. As the story of the carbon budget shows, changes in how the climate issue is enacted do not come only from *outside* established institutions such as the IPCC and the UNFCCC, as challenges to their attempts at stabilizing the issue. They may also originate *within* these institutions, through negotiations and tensions between different groups of actors.

The analysis focuses on the scientific assessment work of the IPCC, starting from the publication of the Fourth Assessment Report (AR4) in 2007, through the production of the Fifth Assessment Report (AR5), and to its publication in 2013–2014. Following the call by Mahony and Hulme (2018) to make the IPCC process itself a central site for investigating the knowledge politics of climate change, I add to the literature seeking to understand the ‘world-making’ powers of the IPCC (Beck and Mahony, 2018; Hulme and Mahony, 2010; Livingston et al., 2018; Sundqvist et al., 2015), by providing a detailed account of how the IPCC process organizes the production of new epistemic objects (Knorr Cetina, 1997) such as the carbon budget.

After setting out my approach to analysing how issues are modified and change over time, I tell the first part of the carbon budget story by contrasting two IPCC reports (AR4 and AR5). In the second part, I expand the analysis to the process of *producing* these reports, by drawing on the documents from which the final reports are constructed (drafts, meeting reports and documents, review comments, and scientific publications), as well as interviews with scientists involved at different levels of the process.^1^ Finally, I discuss the implications of my analysis for the literature on issue formation in STS.

## (Re)enacting issues: Who deserves the credit?

The role of issues as the basis of public participation in democratic politics has become a well-established topic in the STS literature (Callon, 2009; Latour, 2007; Marres, 2005, 2007). In strands of STS associated with actor-network theory (ANT) in particular, the role of issues in mediating public participation around objects or material entanglements as shared ‘matters of concern’ has been highlighted. The interest in issues represents a move towards more explicit engagement with democratic theory in STS (Brown, 2015), but it also links to the field’s long-standing preoccupation with how to open up democratic spaces around the very formation of political problems, that is, the processes that establish what kind of expertise counts, and what kind of questions the political process should seek to answer (e.g. Wynne, 2003). Mapping the public life of issues, therefore, has direct political implications, in that it allows for questions to be asked about who gets to set the terms of democratic decision-making, and how.

The pivotal contribution of Marres is to bring in the American pragmatist tradition of John Dewey and Walter Lippmann, and the way in which they conceived of publics as forming around a specific issue (Marres, 2005, 2007). In their conceptualization, issues and publics emerge as a result of the inability of existing institutions to deal with the matter at hand (e.g. Dewey, 1927: 30–32). A new issue emerges, to borrow an older ANT terminology, as a result of the ‘overflowing’ of existing framings (Callon, 1998): By definition, an issue is that which escapes ‘established political forms’. On this basis, Marres (2007) argues that ‘the issues deserve more credit’ as constitutive of democratic politics.

An important consequence of bringing in this pragmatist understanding of issues and publics is that it allows for an understanding of politics that centres on problematic objects or material entanglements – ‘the matter’ or ‘the trouble to be done away with’ (Dewey quoted in Marres, 2012: 44) – without at the same time reducing politics to technocratic ‘problem-solving’ (cf. Barry, 2013). An issue-centred politics, Marres argues, maintains the constitutive role of conflict posited by radical theorists of democracy (e.g. Mouffe, 2005), as issues emerge precisely through the shortcomings of the problem-solving institutions of ‘liberal instrumentalism’ (Marres, 2012: 56–57).

At the same time, understanding issues as always arising from the shortcomings of existing institutions can easily be taken to imply a specific view of their trajectory: from a situation of institutional failure, which renders the issue problematic and mobilizes a public around it, towards its eventual stabilization and closing-down, as new political agencies are constructed and the issue becomes ‘absorbed by the normal traditions of deliberative democracy’ (Latour, 2007: 817). The danger of this understanding is that issue-politics almost by definition end up being located *outside* of political institutions (Asdal and Hobæk, 2020), and that the *formation* of issues is given priority over analysing how they change over time.

An STS-inspired approach to issue-politics, however, should be careful not to assume any fundamental distinctions between a depoliticizing ‘inside’ and a repoliticizing ‘outside’ of existing institutions. One of the strengths that STS brings to the analysis of politics is a focus on the continuous interactions – struggles, negotiations and alliance-building – that cut across traditional distinctions such as those between stabilized institutions and unruly publics, or between the scientific and the political. Indeed, the expectation would be that it is precisely these interactions that keep issues such as climate change constantly ‘on the move’. In order to capture ‘the historically and geographically contingent ways in which diverse events and materials come to be matters of public dispute’ (Barry, 2013: 8), therefore, an STS analytic of issues needs to account for the role of existing institutions, and to be attentive to how issues are modified and change over time (Asdal, 2015).

Seeking to further expand the literature on issue-politics in this direction, I draw on two existing engagements with the work of Marres. The first is that of Callon (2009; see also Blok, 2014), who suggests paying attention to how issues become specified into distinct ‘problems’, that is, to map processes of ‘problematization’. In line with the classical pragmatist tradition, Callon understands an issue as a ‘situation of initial shock’ stemming from the failure of existing institutions to contain it. Drawing loosely on his earlier use of the term (Callon, 1980), he uses the notion of ‘problematization’ to describe the process through which an issue ‘is gradually being split into a series of distinct problems, some of which are qualified as political and others as economic, technological or scientific’ (Callon, 2009: 543). The term thus presents a way of conceptualizing how struggles over issue articulation may lead towards provisional stabilization, as actors seek to order issues into different epistemic fields and make them manageable for new or existing political agencies.

Second, I follow Asdal’s suggestion to study the ‘little tools’ by which issues are enacted and modified in practice (Asdal, 2008; Asdal and Hobæk, 2020). She proposes the term ‘modifying-work’ (Asdal, 2015) to draw attention to the concrete practices through which issues are shaped and transformed – including the work of material objects, like reports and other paperwork. This presents a ‘down-to-earth approach’ (Asdal, 2008) to following the ways in which issues change over time, which allows the specificity of the issue and the setting in which it is enacted to be foregrounded in the analysis. By focusing on the concrete work and tools of issue-modification, it turns the directionality of change into an open empirical question, thereby going beyond the somewhat schematic trajectory implied by Callon as a movement from the ‘initial shock’ of issue formation towards a stable problematization.

Taken together, these approaches may provide a way to understand the ‘interactional’ life of issues – not as a ‘natural history’ in which they move through clearly identifiable stages from politicization to stabilization, but as ongoing negotiations over their boundaries, meanings and the arrangements through which they should be handled. Identifying the shifting problematizations of an issue provides a way of analysing how it is modified over time, and with what effects. Conversely, paying attention to the concrete practices and specific tools through which modifying-work happens may enable an account of how, in practical terms, actors continuously seek to negotiate and translate unruly and overflowing issues into more manageable problems to be delegated and shifted between institutions – political and scientific.

Employing the analytical strategy elaborated above, I argue that the specific modification of the climate issue enacted by the carbon budget concept has come about not through the emergence of new publics following from a general failure of existing institutions, nor as a result of a fundamental epistemic shift in climate science. Rather, it should be seen as an outcome of the work within existing political and scientific institutions, and the agency they afford scientists and other actors to enact the issue differently. The case of the carbon budget thus suggests that if the literature on issues in STS is to be able to account for how issues are modified over time, it must be open for the fact that such modifications may well take place precisely within the formal institutions – scientific and political – which are sometimes too easily associated with the exact opposite, that is, the closing-down or stabilization of issues.

## The carbon budget as issue modification

In efforts to build a global approach to governing climate change, the IPCC holds a special position (Hulme and Mahony, 2010). Established in 1988 by the World Meteorological Organization and the United Nations Environment Program, it unites virtually all the world’s governments in the effort of producing regular, comprehensive assessments of the scientific knowledge about climate change, its impacts and how to mitigate it. The IPCC’s reports, as well as the increasingly formalized procedures for producing them (Sundqvist et al., 2015), have developed in a process closely entangled with the political process of establishing international mechanisms to regulate greenhouse gas emissions under the UNFCCC (Beck and Mahony, 2018; Miller, 2004; Randalls, 2010).

The publication of the IPCC’s Fourth Assessment Report (AR4) in 2007 is illustrative of the central role the IPCC holds in enacting the climate issue. The report established an authoritative basis of ‘global knowledge’ for the negotiation of a new international climate agreement under the UNFCCC, starting in 2007 and with the aim of finalizing an agreement in Copenhagen, 2009. It also led to the IPCC being awarded, with Al Gore, the Nobel Peace Prize for their work to bring climate change to the attention of political leaders.

As a prime example of the form of authoritative global knowledge that the IPCC brings to the table, the AR4 report is a good starting point for understanding how the carbon budget concept came to modify the climate issue – precisely because the concept, in this report, is completely absent. Rather, the report enacts a climate issue revolving around the concept of ‘climate stabilization’ in the way that this concept itself has been ‘stabilized’ through decades of entangled political/scientific work.

### Targeting a stable climate

AR4 enacts its version of the climate issue largely following the same pattern as previous IPCC reports. First, the report’s division into three parts establishes a specific understanding of the issue’s central problem and its solutions: whereas the report from Working Group I (WGI, on ‘the physical science basis’) lays down the scientific understanding of the climate system, observed changes, and projections of future change, the Working Group II (WGII, on ‘impacts, adaptation and vulnerability’) report describes the consequences of these changes, and Working Group III (WGIII, on ‘mitigation of climate change’) discusses measures and policy responses to address the problem. Second, each report’s ‘Summary for Policymakers’ (SPM) has been approved line by line by government representatives, which, together with other procedures for government involvement, serves to invest the report with a degree of government ownership that helps constitute its authority as global knowledge (Sundqvist et al., 2015). And finally, the Synthesis Report (SYR), which draws together key findings from all three Working Group reports, becomes a condensed expression of the IPCC’s scientific rendering of the climate issue (Livingston et al., 2018).

It is not only through the structure of the report, however, that AR4 enacts the climate issue in a way closely following previous IPCC reports. When the report starts by establishing that ‘warming of the climate system is unequivocal’ (IPCC, 2007: 2) and caused by changes in atmospheric concentrations of greenhouse gases (IPCC, 2007: 5), the key question becomes how to stabilize this concentration. A ‘range of stabilization levels’ is assessed (IPCC, 2007: 19–21), with the temperature rise resulting from them and the emission reductions required to achieve them summarised in a series of figures and tables. The report thus connects directly to the objective of the UNFCCC to achieve a ‘stabilization of greenhouse gas concentrations … at a level that would prevent dangerous anthropogenic interference with the climate system’ (cf. Oppenheimer and Petsonk, 2005).

Enacting the climate issue in these terms raises two scientific problems of fundamental concern: first, in order to know at which level of greenhouse gas concentrations climate change might become ‘dangerous’, one must first know how sensitive the climate system is to changes in atmospheric concentrations. Since the beginning of climate modelling, models were set up to estimate the climate response to CO_2_ emissions at the point when the climate system reached a new state of equilibrium following a doubling of atmospheric CO_2_ concentrations from pre-industrial levels (van der Sluijs et al., 1998). This hypothetical property of the climate system is known as ‘equilibrium climate sensitivity’, and is an important topic of discussion in the WGI report of AR4 (Meehl et al., 2007).

Second, there is the problem of determining an optimal pathway toward stabilizing greenhouse gas concentrations at the desired level. Estimating the volume of annual emissions that would allow for atmospheric concentrations to be stabilized at a certain level requires not only climate modelling, but also economic modelling, since the temporal distribution of emissions is seen as a question of optimization – that is, of achieving stabilization at the lowest cost possible. This is a major topic in the WGIII report of AR4 (Fisher et al., 2007), which presents scenarios from Integrated Assessment Models (IAMs) that quantify greenhouse gas emissions pathways over time that may achieve different stabilization levels (see Cointe et al., 2019).

In this way, the concept of climate stabilization acts as a bridging concept between the different parts of the IPCC report: the concern with equilibrium climate sensitivity of WGI, and the optimization pathways of WGIII are brought together in the Synthesis Report, illustrating how the concept of ‘climate stabilization’ is in fact ‘a ready-made product of science and economics combined’ (Boykoff et al., 2009: 53).

Moreover, the concept serves to bridge not only the different working groups within the IPCC, but also the scientific work of the IPCC and the political work taking place under the UNFCCC, by enacting a very specific ordering of mutually constitutive yet clearly distinguishable scientific and political problems: the scientific problem of determining equilibrium climate sensitivity links to the political problem of determining at what level of atmospheric greenhouse gas concentrations climate change might become ‘dangerous’. Political deliberation on a concentration limit, in turn, allows for the modelling of optimal pathways by which emissions may be distributed temporally, which again enables political discussions about short-term target-setting and commitments to reducing emissions to specific levels within a given timeframe. The climate issue, in this specific problematization, becomes an inseparably political/scientific problem of ‘mitigating and managing the long-term future in a very specific, highly abstract way, since it involves future generations emitting just enough CO_2_ to maintain the concentrations at their target levels indefinitely’ (Boykoff et al., 2009: 55).

The AR4 report, in other words, is thoroughly representative of a climate issue revolving around the concept of ‘climate stabilization’. It represents one way of institutionalizing an issue by breaking it down into specific problems – some designated scientific and some political. These problems can in turn be handled by the international bodies – the IPCC and the UNFCCC – that have been co-produced with this particular enactment of the issue. Notably, built into the focus on climatic stability is also a focus on the stability of climate science itself, which has previously been captured in the notion of ‘anchoring devices’ (van der Sluijs et al., 1998): The reports of the IPCC, including AR4, have worked to continually reaffirm the existing rendering of the climate issue as a way of underwriting its ‘physical science basis’.

### Destabilizing climate stabilization

Given the focus on stability in the IPCC’s reports since the 1990s, it is rather surprising that, a mere five years after AR4 was published, the next IPCC report presents us with a rather different climate issue. In the IPCC’s Fifth Assessment Report (AR5), which was published in several parts over the course of 2013 and 2014, we find a new ordering concept at work, that of the global ‘carbon budget’.

While the structure of AR5 follows that of previous IPCC reports, with the three Working Group reports and a Synthesis Report (SYR) summarizing their main findings, a reading of the SYR reveals a striking difference. After the first chapter has established that the climate system is currently warming due to human-caused greenhouse gas emissions, the second chapter opens with a statement not found in AR4 or previous reports: ‘cumulative emissions of CO_2_ largely determine global mean surface warming by the late 21st century and beyond’ (IPCC, 2014: 56). The chapter further explains that there exists ‘a strong and consistent near-linear relationship’ between global temperature rise and cumulative CO_2_ emissions – that is, the total amount of carbon emitted over all of human history. This means that ‘any given level of warming is associated with a range of cumulative CO_2_ emissions’ (IPCC, 2014: 62). The point is illustrated in a figure (SYR Figure 2.3) which shows a near-linear increase in global temperature as a function of cumulative CO_2_ emissions (IPCC, 2014: 63–64).

The SYR’s focus on cumulative emissions as the central metric of future climate change is substantiated primarily with references to the report from WGI on ‘the physical science basis’, and in particular its 12th chapter about ‘long-term climate change’ (Collins et al., 2013). This chapter elaborates on how cumulative emissions are linearly linked to global temperature increase. In a section titled ‘climate stabilization and long-term climate targets’, the report starts from the concept of stabilization of atmospheric greenhouse gas levels, in line with the ultimate objective of the UNFCCC. It then moves on, however, to note that current policy discussions primarily focus on a temperature target such as 2°C, rather than a target of a specific atmospheric concentration level (Collins et al., 2013: 1107), and highlights the total amount of CO_2_ emitted to the atmosphere as ‘a good indicator’ of when such a temperature target will be reached. In summary:

The simplicity of the concept of a cumulative carbon emissions budget makes it attractive for policy [WBGU, 2009]. The principal driver of long term warming is the total cumulative emission of CO_2_ over time. To limit warming caused by CO_2_ emissions to a given temperature target, cumulative CO_2_ emissions from all anthropogenic sources therefore need to be limited to a certain budget. (Collins et al., 2013: 1112)

Chapter 12 goes into some detail as to how this ‘simple concept’ of a carbon budget is derived, highlighting a number of uncertainties and limitations. Despite acknowledging such complexities, however, the combined message of the SYR and Chapter 12 of the WGI report is clear: Rather than making stabilization of atmospheric greenhouse gas concentrations the central question, as in AR4, one should instead focus on the direct relationship between CO_2_ emissions and temperature in order to calculate the ‘budget’ of total emissions that would be ‘allowed’ in order to keep temperature rise below a certain limit. The carbon budget is explicitly highlighted as a ‘simple’ and ‘more informative’ concept that is ‘attractive for policy’. Moreover, it is rather precisely quantified: the SYR contains a separate table providing specific figures for the amount of CO_2_ that may be emitted before temperature rises by 1.5°C, 2°C or 3°C in different fractions of model simulations, expressed in a manner resembling probability distributions (IPCC, 2014: 64). All of this contributes to making the concept of a cumulative budget of total CO_2_ emissions a key organizing concept in the report.

**Figure 1.**
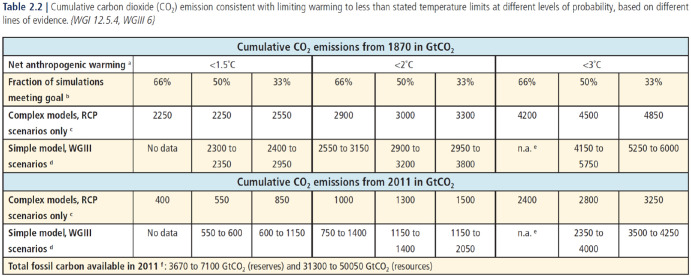
The carbon budgets of AR5, as presented in Table 2.2 of the Synthesis Report (IPCC, 2014: 64).

I should note that AR5 also discusses climate change in terms of atmospheric concentration levels and long-term stabilization. In particular, the report from WGIII is still primarily based on atmospheric concentration levels as the key metric used to categorize different scenarios of future climate change and least-cost emission pathways emerging from IAM modelling (Clarke et al., 2014). The SYR actively attempts to draw the two approaches together, as seen for example in Figure 1 which presents, side by side, budgets derived from ‘complex models’ (i.e. Earth System Models and General Circulation Models used in WGI) and the simple climate model used for the IAM scenarios of WGIII. In this way, the SYR makes clear that the two concepts cannot be seen as mutually exclusive. Rather, they run side by side to a certain extent, with the carbon budget most firmly anchored in WGI and the stabilization concept still dominant in WGIII.

While the distinction between the concepts is not a fundamental one, however, the prominence of the carbon budget in AR5 clearly serves to enact the climate issue in a way that differs from previous IPCC reports. In this way, the carbon budget to some extent *destabilizes* the previous enactment of the issue in terms of ‘climate stabilization’. Whereas the scientific problems following from the climate stabilization concept related to questions of determining equilibrium climate sensitivity and IAM-based modelling of optimal emissions pathways over time, the central problem following from the budget concept is how to determine the precise quantity of ‘allowable’ CO_2_ emissions for given temperature targets. This, in turn, reformulates the problems that the IPCC report leave for the political work taking place in the UNFCCC and elsewhere.

### The political problems of the carbon budget

What, then, is the work being done by the carbon budget concept on how the climate issue is enacted? As seen above, the carbon budget replaces a specific temporal profile of greenhouse gas emissions that prescribe a certain distribution of emissions over time (as found in optimised stabilization pathways) with a single metric of ‘allowable emissions’ as a unitary physical quantity at the global scale. This change in the scientific presentation of the issue simultaneously presents a new set of problems for the political realm. In the discussion of a cumulative emissions budget found in AR5, at least three such problems are present.

First, the existence of a finite amount of emissions that may be ‘allowed’ in order to keep temperature rise below a certain limit means that CO_2_ emissions will have to cease altogether when the budget is exhausted. Chapter 12 of the WGI report explicitly states that stopping temperature from rising above a certain point ‘requires decreasing emissions to near-zero’ (Collins et al., 2013: 1108). Enacting the climate issue as a problem of how to fully eliminate CO_2_ emissions represents a break with previous ideas of determining the optimal level of continued future emissions. The challenge of full decarbonization gestures towards a kind of wholesale societal rearrangement that goes far beyond the more incremental approach of determining the ‘correct’ future emission level. In this sense, the carbon budget casts the climate issue as a more radically transformative one.

Second, by focusing exclusively on CO_2_ emissions and bracketing other greenhouse gases, the carbon budget refocuses the climate issue on the problem of phasing out fossil fuels. The link between the physical quantity of allowable CO_2_ emissions and the comparable physical quantity of remaining fossil fuel reserves is made explicit in SYR Figure 1, which includes an estimate of ‘total fossil carbon available’ – an estimate exceeding the carbon budget for 1.5°C or 2°C many times over. This comparison builds directly on the literature being cited by WGI, in particular Meinshausen and colleagues (2009), which includes the same juxtaposition of allowable carbon emissions and available fossil fuels. In this way, the concept of a cumulative emissions budget explicates a new problem for political attempts to govern climate change, namely how to ensure that large parts of existing fossil fuel reserves are kept in the ground.

Third, the existence of a finite budget of allowable emissions raises the question of how this budget should be allocated – that is, precisely *who* should be ‘allowed’ to take advantage of the remaining atmospheric capacity to store greenhouse gases. In contrast to the two problems described above, this question is only indirectly dealt with in the AR5 discussion of the carbon budget. Among other things, Chapter 12 of the WGI report cites a report by the German Advisory Council on Global Change (WBGU, 2009) in which the distribution of a limited carbon budget among nation-states is a central topic. It is also clear from other literature cited in the WGI report that this question is recognized as an important one (e.g. Raupach et al., 2014; Zickfeld et al., 2009).

Questions of quantified emission limits, distributional effects or restrictions on fossil fuels are in no way new to climate politics. As I noted above, the carbon budget concept does not contradict previous understandings of the issue based on stabilization of a specific atmospheric concentration of greenhouse gases. Nevertheless, the focus on a cumulative emissions budget presents the specific problems highlighted above in new, different or more acute ways. The climate issue is thus *modified* in important respects, with new problematizations to be sorted out by political as well as scientific actors and institutions. The effects of this are evident in the AR5 report, but they are even more clear when examining how the carbon budget concept was received by political actors.

Media reports from the publication of AR5 highlighted the carbon budget as being among the report’s most controversial elements (Harvey, 2013; IISD, 2013), with many governments concerned about its ‘political repercussions’. Versions of the three problems listed above were frequently highlighted: the rapid pace at which emissions would have to be eliminated in order not to exceed the budget; the fact that ‘governments and business may have to leave valuable fossil fuel reserves unexploited’; and ‘how to allocate the remaining “carbon budget” fairly among countries’ (Harvey, 2013). Following the publication of AR5, the same three problems can be traced in a number of political processes, in ways that will not be detailed here. By way of illustration, it can be mentioned that the need to bring emissions to zero is directly reflected in the Paris Agreement, which includes the objective of achieving a ‘balance between anthropogenic emissions by sources and removals by sinks of greenhouse gases’ – that is, ‘net zero’ global emissions – at some time in the second half of the 21st century (Tschakert, 2015).

The radical potential of the carbon budget’s implications regarding a complete phase-out of carbon emissions and leaving fossil fuels in the ground was also seized upon by activists and NGOs. The concept thus became an important underpinning for a radicalization in climate activism, most recently exemplified in the global wave of school strikes under the banner #FridaysForFuture (Fisher, 2019). This is illustrated, for example, in a speech by leading school strike activist Greta Thunberg, in which she refers the audience to a specific page in the latest IPCC report where ‘you will find all our “opinions” summarized. Because there you find our remaining carbon-dioxide budget’ (Thunberg, 2019: 77).

An even clearer illustration of the shift from climate stabilization to carbon budget is provided by American climate activist and author Bill McKibben. McKibben founded the climate activist organization 350.org, which derived its very name from the level at which atmospheric concentrations of CO_2_ should be stabilized. As the IPCC finalized the WGI report of AR5 in 2012, however, McKibben wrote an influential essay in *Rolling Stone Magazine* in which he popularized the concept of the carbon budget as ‘global warming’s terrifying new math’ (McKibben, 2012). The essay, and McKibben’s subsequent ‘Do the Math’ speaking tour, marked a complete shift in focus, where the atmospheric stabilization level of 350 parts per million – despite still being the basis for his organization’s name – was replaced with a focus on the budget for allowable CO_2_ emissions. Here, the new political problematization implied by the carbon budget was presented as a solvable math problem, in which the amount of fossil fuels being extracted would have to be limited in line with the physical constraints of the budget. In this way, much of the surge of activism against fossil fuel development in recent years can be tied directly to the emergence of the carbon budget concept (see also Strauch et al., 2020).

How did this modification of the climate issue come about? In her proposal for an issue-centric approach to politics, Marres emphasizes the pragmatist view that it is the failure of existing political forms to ‘contain the effects of change’ that makes ‘issues appear as an organizing principle of the public’ (2007: 769). With global greenhouse gas emissions still on the rise, the argument may indeed be made that ‘existing political forms’ is failing to address catastrophic climate change, and that this has sparked attempts to re-enact the issue in different ways. Activist efforts to declare a ‘climate emergency’ or to shift the political problem of climate change to questions of fossil fuel extraction are certainly rendered legible in this understanding. But where would this leave the carbon budget? As a discursive attempt to ‘reframe’ the climate issue in terms more amenable to political mobilization, more or less independent of the actual links between the climate system and the carbon cycle that the concept seeks to describe? Or, alternatively, as a scientific breakthrough in the understanding of the climate system, external to the political process associated with the earlier enactment of the climate issue? Neither of these understandings seem satisfactory, for different reasons: in the former case, the content of the carbon budget – the actually existing ‘near-linear relationship’ between emissions and temperature – seems to become lost in discursivity. In the latter, we find ourselves back in an outdated model of an independent sphere of science speaking ‘truth to power’. Can an alternative understanding be developed?

To answer this question, I shift the analytical focus from a close reading of the IPCC’s AR5 report itself, towards the process through which the report was produced. The next section therefore traces the process of producing AR5 and the way in which the carbon budget came to hold such a prominent role within it. Focusing on the tools and procedures of the IPCC process, this approach highlights the work that went into modifying the climate issue, including the interplay – negotiations, translations and sometimes tensions – between actors both within and outside of the IPCC.

## The IPCC process as modifying-work

Publishing an IPCC report is an enormous undertaking, involving hundreds of scientists and government representatives as well as thousands of expert reviewers in an increasingly formalized process spanning several years (Sundqvist et al., 2015). The first stage of preparing a new report is the so-called ‘scoping’ process, in which experts nominated by IPCC member governments work out an outline of each chapter in the new report, which is then approved by governments. The scoping of AR5 took place in 2009, in parallel with preparations for the Copenhagen climate summit – and around the same time as the publication of the first high-profile scientific studies in which a global carbon budget was explicitly quantified.

The documents from the scoping process show that participants were keenly aware of the political context of the report they were preparing. A number of governments, for example, asked for AR5 to provide information about what would be needed to keep temperature below specific thresholds – usually 2°C, which was proposed by the EU and was most widely discussed at the time (Randalls, 2010) – and to inform discussions about UNFCCC Article 2, that is, how to stabilize climate at a level that avoids ‘dangerous anthropogenic interference’ (Livingston et al., 2018: 86). While the interest in knowledge about specific temperature targets was clear, there are no signs of any interest in the carbon budget concept specifically, or more generally in alternative ways of quantifying climate targets or limits. Governments and scientists alike seem to have taken the previous reports’ focus on stabilization of atmospheric concentration levels as a given starting point. It was among the authors of one particular chapter in the WGI report that the idea emerged to give the carbon budget concept a prominent role.

### Establishing a ‘physical science basis’

The report of WGI deals with the ‘physical science basis’ of climate change, that is, the scientific knowledge about the climate system in its past, present and future states. Of particular interest for climate policy discussions are the report’s long-term climate projections based on complex climate and Earth system models, which describe how the climate is likely to change in response to human influence. In AR5, this topic was covered in Chapter 12 of the WGI report. When the work on this chapter started, the long-term climate modelling had generally changed little since previous reports, and the chapter authors were therefore looking for ways in which the report could contribute something ‘new’ in order to be seen as ‘relevant’ (interviews). Some of the authors had been centrally involved in studies that quantified carbon budgets for 2°C, and suggested that the concept represented such a contribution. While other participating scientists did not initially see the carbon budget as particularly important, they had no objections to highlighting it as a way of ensuring the ‘novelty’ that could increase the report’s relevance (interviews). The concept responded directly to governments’ interest in the temperature targets that had become an important political question in the UNFCCC negotiations, with the EU pushing for a 2°C limit and many developing countries favouring stricter limits of 1°C or 1.5°C (Tschakert, 2015). And most importantly, it had already become established in the scientific literature.

As the IPCC’s role is to assess existing knowledge, its reports are supposed to build exclusively on previously published results. For the report of the IPCC’s WGI to lay out the ‘physical science basis’ of climate change, therefore, the report itself needs a ‘physical basis’ in the established scientific literature. As the case of the carbon budget shows, however, the development of this literature cannot be understood independently of the tools and procedures of the IPCC process. In part, the emergence of the literature that enabled the carbon budget’s central role in AR5 can itself be traced back to the IPCC, and the modelling work that had been undertaken for the two previous IPCC reports.

From 2008–09 onwards, a number of new studies had been published that quantified the cumulative CO_2_ emissions associated with different levels of temperature rise. Probably the most high-profile publications were two articles in the journal *Nature* in April 2009, one from a team led by Myles Allen at the University of Oxford, and one led by Malte Meinshausen at the Potsdam Institute for Climate Impact Research (Allen et al., 2009; Meinshausen et al., 2009). However, several similar articles were published around the same time by scientists in Canada and the UK (Anderson and Bows, 2008; Matthews et al., 2009; Zickfeld et al., 2009). What these studies did was, in effect, to combine the expectation that long-term climate targets would be based on the EU-backed 2°C temperature limit, with an increasingly explicit scientific understanding that there is an almost linear relationship between cumulative CO_2_ emissions and global temperature rise.

The near-linear relationship between CO_2_ and temperature has been established through modelling that links the carbon cycle and the climate system. In each of these systems, there are mechanisms that, if seen in isolation, render the relationship non-linear: In itself, the warming effect of a unit of CO_2_ added to the atmosphere declines as the atmospheric CO_2_ concentration increases, because the radiative absorption bands of CO_2_ gradually become saturated. At the same time, the ocean’s ability to absorb CO_2_ from the atmosphere declines as carbon concentrations and temperatures rise, gradually leaving a larger fraction of CO_2_ emissions in the atmosphere. When modelled together, however, these two unrelated processes roughly cancel each other out, resulting ‘by chance’ (Zickfeld et al., 2016: 2) in a near-linear relationship between CO_2_ emissions and temperature. The consequence of this ‘chance linearity’ is a major simplification in how human influence on the climate system can be understood: Each new unit of CO_2_ added to the atmosphere will add a comparable unit of warming, regardless of when emissions take place. From this insight, it follows that the main determinant of CO_2_’s effect on temperature is the total stock of CO_2_ in the atmosphere, that is, cumulative emissions. It also follows that if temperature rise is to be brought to a halt, so must emissions.

The way that feedbacks between the climate system and the carbon cycle produce an almost linear relationship between CO_2_ and temperature was shown already in the early 1990s (Caldeira and Kasting, 1993). In 2008, H Damon Matthews and Ken Caldeira built on this older study to show that CO_2_ emissions effectively would have to cease completely if temperature was to be stabilized at any level (Matthews and Caldeira, 2008). Matthews, Kirsten Zickfeld and others built further on this to highlight the proportional relationship between warming and cumulative CO_2_ (Matthews et al., 2009) and calculate cumulative emissions for given temperature limits (Zickfeld et al., 2009), publishing their results around the same time as Meinshausen, Allen and colleagues.

The carbon budget literature, in other words, was a product of several teams working more or less in parallel on the same topics. In interviews, scientists involved in the process describe it in similar terms, as ‘an interesting story about how an idea sort of *emerges* from the scientific community’ after it ‘had sort of been kicking around’ for a while. It is clear, however, that this ‘emergence’ was contingent on a number of factors, one of them being political debates over temperature targets in the UNFCCC. Another factor was the IPCC process itself: The modelling work that formed the basis for AR4 in 2007 became an important basis for further work in the following years (e.g. Solomon et al., 2009). Specifically, the WGI report of AR4 had noted that models that included feedbacks between the carbon cycle and the climate system showed a reduced ocean carbon uptake, which had implications for the cumulative emissions associated with a given climate stabilization target (IPCC, 2007: 67; Meehl et al., 2007: 791). Although AR4 did not explicitly show that this feedback resulted in a near-linear relationship between CO_2_ and temperature, the report’s discussion of cumulative emissions provided the impetus for the study in which Kevin Anderson and Alice Bows-Larkin (2008) calculated a carbon budget for the 2°C limit the following year.

The ‘emergence’ of the carbon budget literature thus highlights the organizing role of the IPCC: In bringing together scientists to assess existing knowledge, the IPCC process simultaneously establishes a set of relations and expectations that result in new knowledge being produced. By facilitating the accumulation of modelling data through large-scale ‘model intercomparison’ initiatives, the IPCC establishes a common infrastructure for further work, as well as shared understandings of knowledge ‘needs’ and ‘gaps’ (cf. Hulme, 2018) that specify the problems toward which the work should be directed.

### Targeting modified targets

While the role of the IPCC process in enabling new scientific problematizations was one of the factors behind the emergence of the carbon budget concept, several contributors to the carbon budget literature also had very explicit *political* aims, clearly targeting a change in the way climate policy targets are understood and discussed. Myles Allen, David Frame and others had already argued for several years that the existing focus on climate stabilization was a problem for climate policymaking, because it relied on the persistently uncertain entity of ‘equilibrium climate sensitivity’ (e.g. Allen et al., 2006). Their work to develop alternatives resulted in the 2009 article, where they argued that, on a general level, ‘policy targets based on limiting cumulative emissions of carbon dioxide are likely to be more robust to scientific uncertainty than emission-rate or concentration targets’ (Allen et al., 2009: 1163).

Others placed their work even more directly into the political context prior of the Copenhagen climate conference and ongoing discussions about a 2°C warming limit. This was the starting point for the article by Malte Meinshausen and colleagues (2009), which pointed to more than 100 countries having already adopted this limit; and for Kevin Anderson and Alice Bows-Larkin (2008), who calculated cumulative emissions budgets as input into UK policy discussions. Current climate policy, they argued, was ‘dominated by long-term reduction targets’ without regard for the ‘cumulative carbon budget’ leading up to those targets (Anderson and Bows, 2008: 3865). In this way, the carbon budget became a way of making visible the effect of action in the short-term on long-term targets, modifying the temporality of the climate issue so as to ‘bring both the distant past and the distant future into view’ (Frame et al., 2014: 692) through a single number of ‘allowable’ emissions.

Not all scientists shared the view that carbon budgets provided a politically useful way to think about the temporality of climate change, however. Ken Caldeira, who had been among the first to establish the near-linear relationship between CO_2_ and temperature, explicitly rejected the political implications of the ‘budget’ term, warning that describing some amount of emissions as ‘allowable’ would simply encourage policymakers to ‘let the guys down the road deal with it when the budget has been exceeded’ (Romm, 2013). Some of the scientists involved in developing the concept were also aware of potential downsides in policy discussions. For example, as the article by Meinshausen and colleagues was published in 2009, the press material accompanying it indicates concern that introducing a new concept so close to the Copenhagen climate conference could be seen as disturbing or upsetting the delicate negotiation process (PIK, 2009).

Such concerns and disagreements highlight that the political implications of their work were discussed and reflected upon by scientists developing the carbon budget concept. It also draws attention to how the connotations of the term ‘budget’ are read differently by different actors, and to the term’s longer history in climate science and policy. The notion of a ‘budget’ was already used in some contexts to refer to emission reduction targets such as those established under the Kyoto Protocol, or nationally as in the UK Climate Change Act. Even the more precise term ‘carbon budget’ was already in use in the 1980s (e.g. Krause et al., 1989; see also Lahn, 2020). These earlier uses were well known by at least some of the scientists that contributed to the new carbon budget literature (see PIK, 2009). However, they also differed from how the term was used in the literature that emerged in 2008–2009, in that the budget was seen more as a policy tool than a scientific concept (Lahn, 2020). Because it was not linked to the understanding that the relationship between CO_2_ and temperature is nearly linear, it was assumed that the temporal distribution of carbon emissions was essential to determining their effects on climate. Therefore, a carbon budget was only seen as an ‘approximation’, because cumulative emissions for a given warming limit was expected to vary depending on their distribution over time (Krause et al., 1989: 37–42). The timing of emissions, in turn, was a question of economics – a matter of optimizing resource use – and therefore required assumptions drawn from scenario building and economic modelling (Randalls, 2011).

While the new scientific literature in 2008–09 clearly drew on earlier notions of budgeting as a policy tool, it simultaneously worked to re-categorize the concept as a fundamentally scientific one, understood as a physical constraint derived from climate system modelling. The new understanding of a near-linear relationship between carbon emissions and temperature response was crucial to this re-categorization: Rather than being seen as an ‘approximation’ with inherent scientific problems, the carbon budget reappeared as a concept ‘purely based on Working Group I science’ (i.e. physical science), which required – in the enthusiastic words of one WGI scientist – ‘no assumptions on scenarios, no economics, no optimization, nothing’ (interview). In this way, the ‘budget’ term was repurposed to describe a physical limit, paradoxically ridding the concept of the economics from which the budgeting metaphor was originally drawn.

To sum up: The publications about a cumulative emissions budget that appeared from 2008 to 2009 together established a scientific literature underpinning the carbon budget concept. This literature was built on a combination of, on the one hand, an improved understanding of the links between the climate system and the carbon cycle that developed out of earlier work connected to the IPCC process, and on the other, the explicit aim of some scientists to find new ways of formulating climate policy targets, drawing on earlier use of the budget notion. It was the production of this scientific literature that allowed the carbon budget to become established as a physical-science concept and given a lead role in Chapter 12 of the WGI report, where Myles Allen, Malte Meinshausen and Kirsten Zickfeld were among the contributing authors. This, in turn, was crucial for bringing the concept back into the political arena: When scientists presented the *Nature* papers for negotiators in the UNFCCC in 2009, they were told that the findings would have to ‘go through the IPCC process’ before they could really be considered by policymakers (interview). For the purpose of intervening in policy target formulation, in other words, establishing a scientific literature is not enough. The literature also needs to pass through the obligatory passage-point of the IPCC process and be reproduced in a specific form: the IPCC report.

### From literature to report – line by line

Once the concept was established in the scientific literature, its proponents could bring it into Chapter 12 of the AR5 WGI report. And once it was featured in Chapter 12, it could become included in the report’s more widely read Summary for Policymakers (SPM). At this stage, however, it encountered resistance, as the WGI report was submitted to governments for final acceptance. In September 2013, government delegates met to accept the WGI report and go through the formalized IPCC procedure of approving the report’s SPM ‘line by line’. In this process, the carbon budget proved to be among the most controversial topics, and was, according to reports from the meeting, the last part of the SPM to be approved (Harvey, 2013; IISD, 2013). In particular, large developing countries like China, Saudi Arabia and Brazil opposed the reference to cumulative emissions (IISD, 2013). Among other things, a figure illustrating the near-linear relationship between CO_2_ and temperature was criticized for placing all of the focus on CO_2_, while ignoring other greenhouse gases.

The authors of Chapter 12 were to some extent prepared for such resistance. Comments received in the review process, as well as ‘rumours’, had alerted them to the fact that some governments found the focus on cumulative emissions controversial (interviews). The authors had anticipated this by making sure that each sentence of the text in Chapter 12 was backed up by an abundance of references to the scientific literature, so as to underscore the scientific basis of their claims (interview). Furthermore, the scientists participating in the approval meeting were in continuous contact with other carbon budget scientists who could compile modelling data ‘on the fly’ to provide the meeting with updated numbers whenever government representatives asked for additional information (interview). Discussions between chapter scientists and government delegates lasted into the early hours of the morning of the approval meeting’s final day. Approaching the deadline, and the scheduled press conference to present an adopted SPM, the countries who had objected eventually gave in, and the section on cumulative emissions was accepted in the SPM of Working Group I with some additions (interviews; Harvey, 2013).

Having been successfully made part of the SPM of WGI, the concept could then be given an even more central role in the Synthesis Report (SYR) of AR5, which was published the following year. The SYR draws together the most policy-relevant findings from all three Working Group reports. The report highlights cumulative emissions of CO_2_ as the most important among the ‘key drivers of future climate’ (IPCC, 2014: 56). The concept was backed up by the figure that had previously caused controversy in the approval of the WGI SPM, as well as a brand new table that presented an overview of the size of the carbon budgets presented both in Working Group I and Working Group III (Figure 1, see above). The table was produced specifically for the SYR, and served to further highlight the concept of cumulative emissions. Again, some of the scientists who had been central in developing the carbon budget literature, like Myles Allen, were directly involved in the IPCC work as part of the SYR ‘core writing team’. And again, they were able to draw on other scientists to help produce the numbers that would be needed for the table – much as in the process of government approval of the WGI SPM (interviews).

Through the process of government review and approval of the SYR, the table in Figure 1 was shaped by the demands of policymakers in several ways. Most notably, some developing countries that favoured a lower temperature limit than 2°C in the UNFCCC negotiations asked for the IPCC to include budgets for a temperature target of 1.5°C (interviews; Tschakert, 2015). Budgets for 1.5°C had not been assessed to a significant degree in the published literature, which had largely taken the EU proposal of 2°C as a starting point. The numbers for this temperature target therefore had to be constructed specifically for the table based on models that were originally designed for higher temperature targets. Although these numbers therefore had a weak basis in the scientific literature, they were included for fear that some developing countries would otherwise oppose the table being included in the SYR (interviews).

In the end, the table was accepted without controversy in the section-by-section adoption of the SYR (IISD, 2014) – to some surprise among the scientists participating in the process (interviews). Even the bottom row of the table, which compares available carbon budgets with the amount of available fossil fuel resources, was accepted by government delegates – leaving scientists to speculate that some governments, like that of Saudi Arabia, had not been paying sufficient attention (interviews). Figure 1, although produced in a rather ad-hoc manner specifically for the AR5 SYR, became a definitive expression of the scientifically established ‘carbon budget’ that had been gradually developed in the literature over the previous years. With its explicit contrast between the world’s remaining budget and the amount of CO_2_ stored in remaining fossil fuel reserves, the table willingly lent itself to political statements about the need to leave coal and oil in the ground, and the imperative of rapid decarbonization.

### The little tools of the IPCC process

With the WGI report featuring the concept both in Chapter 12 and the SPM, and the SYR report giving it an even more prominent role as a key determinant of future climate, the publication of AR5 firmly established the carbon budget as a new, central concept for understanding climate change. As the above analysis of the IPCC process makes clear, this modification of the climate issue was not something the member governments of the IPCC set out to achieve. It was rather the result of conscious efforts from a group of scientists who had first developed the concept in the scientific literature, and then moved it step by step into the AR5 process, in order to enact the issue in a way they saw as more amenable to political action. The IPCC process was itself the condition of possibility for this work: By establishing networks of scientists and data sets from earlier modelling work, it provided an infrastructure through which a new scientific literature about the near-linear relationship between CO_2_ and temperature could emerge.

More than simply assessing existing knowledge, therefore, the IPCC process arguably helped produce a new object of scientific inquiry. It also provided a setting for presenting this object to policymakers and negotiate its specific presentation so as to invest it with authority and legitimacy. Such negotiations could even take the form of producing completely new numbers to assuage the concerns of governments, as in the case of the AR5 SYR table mentioned above. In the community of contributing scientists, there exists a shared ambition to be *policy-relevant*, and a perception that each new IPCC report needs to present novel results or original framings in order to demonstrate such relevance (cf. Livingston et al., 2018). This perception clearly contributed to the carbon budget concept being given a prominent role in AR5 – not despite its absence in previous assessment reports, but rather because of it.

In this sense, both the formalized procedures and the more informal expectations which govern the IPCC process can be seen as tools that afford scientists the means to change how the climate issue is enacted: The scoping process alerted authors to the possibility to intervene in policy discussions about global temperature targets, and the approval process, in which figures and tables are adjusted so as to become acceptable to different political actors, allowed for the scientific literature on the carbon budget to be translated into an authoritative form. In the published report, these tables and figures can be seen as little tools in their own right, subtly changing how climate change is enacted as a political issue by pointing out that emissions will have to be brought to zero for temperature rise to stop, or that existing fossil fuel reserves already exceed the global budget of ‘allowable emissions’.

## Conclusions: Changing climate change

The IPCC’s enactment of the climate issue changed markedly from AR4, which was dominated by the concept of ‘climate stabilization’, to AR5, in which the carbon budget concept was given a prominent role. This change was made possible by the IPCC process itself, with its continuous interaction between new scientific work and institutional expectations about novelty and ‘policy-relevance’, as well as by some scientists’ explicit pursuit of specific changes in the political parameters of the climate issue. Through this process, the IPCC was able to establish an authoritative understanding of what is known about the carbon budget, where there are still ‘gaps’ and therefore scientific problems, and, crucially, what political problems are left for governments and institutions such as the UNFCCC to address. The political stakes of this change were clearly understood by governments participating in the IPCC process, as shown by the controversy that arose in the approval of the WGI report.

To understand this change in how the climate issue is enacted, the notion of problematization is helpful, as it directs attention to how the IPCC process serve to delineate between what is taken for granted, what is known and unknown, producing distinct climatic ‘problems’ to replace or augment existing ones and distributing them between different institutions and social spheres. A new problematization ‘does not necessarily attack previously fabricated knowledge or established theoretical systems’ (Callon, 1980: 206): The concept of the carbon budget does not represent a complete break with the earlier focus on long-term stabilization of atmospheric greenhouse gas concentrations, whether scientifically or politically. Rather, it builds on and goes beyond established climate science and policy targets to suggest new problems on which to focus. In this way, the issue itself is modified: The carbon budget presents us with a climate issue that is different from that of long-term stabilization targets. This, in turn, has political effects – such as the adoption of new targets to reach ‘net zero’ greenhouse gas emissions, or activist campaigns to leave fossil fuels in the ground.

Callon stresses the ‘creative capacities of actors’ to engage in struggles over problematization (Callon, 2009: 545). In the history of the carbon budget, the explicit goals of some scientists to change the way climate targets are set certainly played an important role. However, the analysis here also brings out another aspect that is less clearly present in Callon’s account – and indeed in much of the literature on issue formation: namely the institutional setting of the IPCC, and the capacities it affords scientists to modify the climate issue. Actors with strong views about the kind of targets that would be most conducive to political action on climate change were helped in their efforts not only by their own creative capacities, but also by the ‘little tools’ (Asdal, 2015) of the IPCC and its interactions with the UNFCCC process. These tools, found both in the formalized process of producing reports (its procedures for involving governments and conferring legitimacy, its expectations about novelty and policy-relevance) and in the reports themselves (the figures and tables through which they give a scientific literature an authoritative and ‘usable’ form) helped shape the carbon budget concept, providing scientists the means by which they were able to enact the climate issue differently.

Focusing on the specific tools by which the climate issue is modified, in other words, serves to highlight the institutional setting in which modifying-work takes place. It thereby underscores the historical contingency of how struggles over problematization play out. It also calls into question the assumption, drawn from the classical pragmatist tradition, that issue formation is necessarily a process characterized by the failure of established institutions to contain the issue. There may well be reasons to question the ability of institutions such as the IPCC and the UNFCCC to adequately deal with the climate issue, and therefore to look outside these institutions for political challenges that seek to redefine climate change as a matter of concern. The history of the carbon budget, however, shows that such modifications can also originate within such institutions. As part of their ordinary practices of producing reports and staging meetings between scientists and government representatives, these institutions can become sites for re-working and re-enacting issues. Attending to these sites, and how they enable new problematizations to be negotiated and translated between actors both within and outside of their institutional setting, promises to further open up the processes that shape issue-politics.

The analysis above thus suggests that the literature on issues in STS should be attentive to how issues are modified over time, and that not only the issues themselves, but also the institutions that attempt to deal with them, deserve more credit and scholarly attention. This can help ensure that the shifting and multivalent forms of a highly complex issue such as climate change is not slipping away from analysis, but that we are instead alert to how climate change itself changes – and to the political implications of such change.

## Notes

1. Drafts and review comments for the AR5 report, as well as official documents from IPCC meetings, were obtained from the IPCC website, https://archive.ipcc.ch. Reports from IPCC meetings are published by IISD in *Earth Negotiations Bulletin*. Additionally, seven in-depth interviews with scientists affiliated with the IPCC and/or publishing on the topic of carbon budgets were conducted between January 2018 and January 2019.
